# Early Decline in Glucose Transport and Metabolism Precedes Shift to Ketogenic System in Female Aging and Alzheimer's Mouse Brain: Implication for Bioenergetic Intervention

**DOI:** 10.1371/journal.pone.0079977

**Published:** 2013-11-11

**Authors:** Fan Ding, Jia Yao, Jamaica R. Rettberg, Shuhua Chen, Roberta Diaz Brinton

**Affiliations:** 1 Department of Pharmacology and Pharmaceutical Sciences, School of Pharmacy, University of Southern California, Los Angeles, California, United States of America; 2 Department of Neurology, Keck School of Medicine, University of Southern California, Los Angeles, California, United States of America; University of Pécs Medical School, Hungary

## Abstract

We previously demonstrated that mitochondrial bioenergetic deficits in the female brain accompanied reproductive senescence and was accompanied by a shift from an aerobic glycolytic to a ketogenic phenotype. Herein, we investigated the relationship between systems of fuel supply, transport and mitochondrial metabolic enzyme expression/activity during aging (3–15 months) in the hippocampus of nontransgenic (nonTg) background and 3xTgAD female mice. Results indicate that during female brain aging, both nonTg and 3xTgAD brains undergo significant decline in glucose transport, as detected by FDG-microPET, between 6–9 months of age just prior to the transition into reproductive senescence. The deficit in brain metabolism was sustained thereafter. Decline in glucose transport coincided with significant decline in neuronal glucose transporter expression and hexokinase activity with a concomitant rise in phosphorylated/inactivated pyruvate dehydrogenase. Lactate utilization declined in parallel to the decline in glucose transport suggesting lactate did not serve as an alternative fuel. An adaptive response in the nonTg hippocampus was a shift to transport and utilization of ketone bodies as an alternative fuel. In the 3xTgAD brain, utilization of ketone bodies as an alternative fuel was evident at the earliest age investigated and declined thereafter. The 3xTgAD adaptive response was to substantially increase monocarboxylate transporters in neurons while decreasing their expression at the BBB and in astrocytes. Collectively, these data indicate that the earliest change in the metabolic system of the aging female brain is the decline in neuronal glucose transport and metabolism followed by decline in mitochondrial function. The adaptive shift to the ketogenic system as an alternative fuel coincided with decline in mitochondrial function. Translationally, these data provide insights into the earliest events in bioenergetic aging of the female brain and provide potential targets for preventing shifts to less efficient bioenergetic fuels and transition to the ketogenic phenotype of the Alzheimer's brain.

## Introduction

Previously we demonstrated that loss of ovarian hormones and reproductive senescence (9–12 months) during female aging were associated with a significant decline in aerobic glycolysis and mitochondrial oxidative phosphorylation as well as decreased activities of key bioenergetic enzymes, pyruvate dehydrogenase (PDH) and Complex IV cytochrome c oxidase (COX) [1]–[4]. The decline in mitochondrial bioenergetics was exacerbated in the female triple transgenic Alzheimer's (3xTgAD) mice and preceded the development of mitochondrial β-amyloid (Aβ oligomer) at 9 months of age and thereafter [4]. During the transition to reproductive senescence, COX activity decreased by 40% in both the wild type (nonTg) and 3xTgAD brains which is predictive of a decline in ATP generation [2]. The decline in PDH activity was accompanied by a significant increase in enzymes required for ketone body utilization (3-oxoacid-CoA transferase 1, SCOT) and long-chain fatty acid metabolism (hydroxyacyl-Coenzyme A dehydrogenase/3-ketoacyl-Coenzyme A thiolase/enoyl-Coenzyme A hydratase (trifunctional protein), HADHA), which are indicative of compensatory fuel utilization [2]. These bioenergetic changes observed during reproductive senescence were recapitulated in the ovariectomized rodent model of human menopause [3]. Consistent with basic science findings, data emerging from clinical positron emission tomography with 2-[^18^F]fluoro-2-deoxy-D-glucose (FDG-PET) analyses demonstrate a significant decline in cerebral glucose metabolic rate (CMRglu) in the posterior cingulate (PCC) in postmenopausal women [5] and a decline in cognition during the perimenopause transition in women [6], [7].

Glucose hypometabolism and a shift to alternative substrates have been identified as a metabolic phenotype characteristic of the Alzheimer's brain [8]–[13]. FDG-PET imaging analyses have revealed a significant decline in CMRglu, particularly in posterior cingulate (PCC) and parietal-temporal cortex, in persons with Alzheimer's disease as well as those at increased risk for AD [9]–[13]. Multiple FDG-PET imaging studies in neurologically normal persons who are at risk for AD, persons with an ApoE4 genotype [12], [13] and persons with maternal history of AD [14], present with significant hypometabolism in brain prior to development of pathology. Further, in persons with AD, brain glucose hypometabolism is accompanied by the activation of alternative metabolic pathways, as evidenced by a utilization ratio of 2∶1 glucose to alternative substrate in persons with incipient AD compared to a ratio of 29∶1 in healthy elderly controls [8].

As described above, our previous findings indicated a significant decline in the bioenergetic system of the female brain that coincided with its transition through reproductive senescence. Human reproductive senescence is characterized by three stages, perimenopause, menopause and postmenopause [15], [16]. The reproductive transition in rodents occurs within a well-defined time frame and shares multiple features and endocrine changes found in human perimenopause such as a decline in follicles, irregular cycling and irregular fertility [15], [17], [18]. Cycle irregularity is a well-characterized indicator for the onset of reproductive transition in both rodents and humans [15], [17], [18]. Previous reports identified that cycling irregularity occurs in female mice between 7 and 12 months of age as evidenced by change in both cycle frequency and length [19].

Based on our earlier findings that the female aging brain developed deficits in aerobic glycolysis and mitochondrial respiration during reproductive senescence that were followed by increased expression of enzymes required for long-chain fatty acid (HADHA) and ketone body (SCOT) metabolism [2], [4], we investigated the expression of substrate transporters during the reproductive senescence transition. Specifically, we determined whether age-related decline in the bioenergetic system of the brain was associated with changes in glucose uptake, substrate (glucose, lactate/ketone body) transporter expression and/or enzyme systems required for glycolysis. Further, we sought to determine whether there was a shift to the utilization of alternative fuels, the adaptive mechanism in response to the decline in brain bioenergetics. Outcomes of the current study indicate an early and significant impact of female aging on the substrate transporter systems of the brain, which appear to be initiating events that lead to dysfunctional bioenergetic system in brain.

## Materials and Methods

### Animal Treatments and Ethics

All rodent experiments were performed following National Institutes of Health guidelines on use of laboratory animals and an approved protocol (protocol number: 10217) by the University of Southern California Institutional Animal Care and Use Committee. The presented study has been approved by the University of Southern California Institutional Animal Care and Use Committee (Ethics Committee).

### NonTg and Transgenic mice

Colonies of the 3xTgAD mice strain (129S; Gift from Dr. Frank Laferla, University of California, Irvine) [20] were bred and maintained at the University of Southern California (Los Angeles, CA) following National Institutes of Health guidelines on use of laboratory animals and an approved protocol by the University of Southern California Institutional Animal Care and Use Committee. Mice were housed on 12 hours light/dark cycles and provided ad libitum access to food and water. The characterization of amyloid and tau pathologies, as well as synaptic dysfunction in this line of mice has been described previously [20] and confirmed in our laboratory. Mice were genotyped routinely to confirm the purity of the colony. To ensure the stability of AD-like phenotype in the 3xTgAD mouse colony, we performed routine immunohistochemical assays every 3 to 4 generations. Only offspring from breeders that exhibit stable AD pathology were randomized into the study. Only intact female mice at the age of 3, 6, 9, 12 and 15 months were used for the experiment.

### microFDG-PET and microCT imaging

Mice were maintained under anesthesia during microPET and microCT scans with 2–2.5% isoflurane in oxygen. Anesthesia is the standard procedure for small animal imaging to ensure that body movement does not occur which would nullify the images acquired. To ensure that any effect of anesthesia on brain glucose uptake were controlled for/distributed across all ages and genotypes, all imaging analyses were conducted with mice from each age group.

Scans were performed in an imaging chamber equipped with a nose cone for anesthesia delivery and heating pad for body temperature control. MicroPET imaging was performed with a microPET R4 rodent model scanner (Concorde Microsystems Inc, Knoxville, TN) and micro CT imaging was performed on MicroCAT II tomography (Siemens Preclinical Solutions, Knoxville, TN). In our mouse microPET scan, the impact of skull thickness on the signal is very small. For microPET imaging (FDG: 511 keV), the linear attenuation coefficients of bone and water are 0.192/cm and 0.096/cm. The skull thickness of the mouse is usually 0.5 mm, and the attenuation factor is 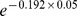 = 0.990. If we have 0.5 mm of soft tissue, the attenuation coefficient of which is similar to water, this factor becomes 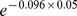 = 0.995. Thus the difference in attenuation coefficient between skull and soft tissue is smaller than 1% ((0.995–0.990)/0.990 = 0.51%).

Mice were injected intravenously via the tail vein with radiotracer [^18^F] Fluoro-2-deoxy-2-D- glucose (FDG, 200 µCi, 100 µL). The dosage of FDG is Radioactive dose was determined prior to injection by radioisotope dose calibrator (Capintec, CRC-712M). At 40 min post-injection of FDG, each mouse was positioned in the MicroPET scanner in the center of the 10.8 cm transaxial and 8 cm axial field of view (FOV). Brain microPET data were collected for 10 min followed by a 10 min microCT scan for the purpose of co-registration.

Co-registration of microPET and microCT data was performed using the AMIDE software package (http://amide.sourceforge.net/). After co-registration of the PET and CT images, ROI (region of interest) was defined and used to measure the radioactivity concentration in brains. Decay correction was used to adjust the actual radioactivity dosage injected (Actual radioactivity dosage at time of injection = Initial radioactivity × 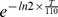, T  =  T minutes between injected time point and initial time point).

### Brain tissue preparation and Western blot analysis

Upon completion of FDG-MicroPET imaging, mice were sacrificed and the brains rapidly dissected on ice. Hippocampus was processed for protein extraction using Tissue Protein Extraction Reagent (Thermo Scientific, Rockford, IL, USA) with phosphatase and protease inhibitors (Sigma, St. Louis, MO, USA), and protein concentrations were determined with the Bio-Rad Bradford assay. Equal amounts of protein (20 mg/well) were loaded in each well of a 12.5% SDS PAGE Criterion gel (Bio-Rad, Hercules, CA) and electrophoresed with Tris/glycine running buffer (pH 8.3). Proteins were transferred to 0.45 mm pore size polyvinylidene difluoride (PVDF) membranes and immuneblotted with GLUT1 (glucose transporter 1) antibody (1∶1500, Abcam, Cambridge, MA, USA), GLUT3 (glucose transporter 3) antibody (1∶1000, Abcam, Cambridge, MA, USA), Hexokinase II antibody (1∶1000, Millipore, Billerica, MD, USA), MCT1 (Monocarboxylate transporter 1) antibody (1∶1000, Millipore, Billerica, MD, USA), MCT2 (Monocarboxylate transporter 2) antibody (1∶1000, Millipore, Billerica, MD, USA). HRP-conjugated anti-rabbit antibody and HRP-conjugated anti-mouse antibody (Vector Laboratories, Burlingame, CA, USA) were used as secondary antibodies. Immunoreactive bands were visualized with Pierce SuperSignal Chemiluminescent Substrates (Thermo Scientific, Waltham, MA, USA) and captured by Molecular Imager ChemiDoc XRS System (Bio-Rad Laboratories, Hercules, CA, USA). All band intensities were quantified using the Un-Scan-it (version 6.0, Silk Scientific, Orem, UT, USA) software.

### Hexokinase activity assay

Hexokinase activity assay was measured by monitoring the conversion of NAD+ (nicotinamide adenine dinucleotide) to NADH (reduced nicotinamide adenine dinucleotide) by following the change in absorption at 340 nm. The assay medium contained: 0.1 mg/mL of the hippocampal tissue protein, 0.05 M Tris/HCl, pH 8.0, 13.3 mM MgCl_2_, 0.112 M glucose, 0.227 mM NAD+, 0.5 mM Adenosine 5′Triphosphate, and 1 IU/mL glucose-6-phosphate dehydrogenase (*Leuconostoc mesenteroides*) in a final volume of 150 mL. The OD at λ = 340 nm was measured every 1 min for 30 mins at a temperature of 30 °C. The increase in OD reflects the increase in NADH concentration, and the total hexokinase activity was calculated from the slope of the resulting curve.

### Immunohistochemistry

For immunohistochemistry studies, fixed hemispheres were coronally sectioned at 30 mm, and then processed for immunohistochemistry using a standard protocol. Briefly, every 12^th^ section was blocked (1 h at RT, PBS with 5% goat serum and 0.3% trinton x-100), immunolabeled using antibody directed against Ab for (PhosphoPDH, Covance, 1∶1000 dilution 4°C overnight), followed by washing and secondary antibody Fluorescein goat anti-mouse (1∶500, Chemicon, Ramona, CA, 1 h at RT) and/or Cy3 conjugated goat anti-rabbit (1∶1000, Chemicon, Ramona, CA, 1 h at RT). Sections were mounted with anti-fade mounting medium with DAPI (Vector Laboratories, Burlingame, CA). Antigen unmasking treatment, consisting of 5 min rinse in 99% formic acid was performed to enhance Ab immunoreactivity (IR). Fluorescent images were taken using a fluorescent microscope with the slide book software (Intelligent Imaging Innovations Inc, Santa Monica, CA).

### Plasma β-hydroxybutyric acid measurement

The concentration of plasma β-hydroxybutyric acid was determined by β-hydroxybutyrate liquicolor assay kit (Stanbio laboratory, TX, USA) following manufacturer's instructions.

### Statistic analysis

Statistically significant differences between groups were determined by an ANOVA followed by a Newman–Keuls post-hoc analysis. Statistical significance of the correlation was determined by Pearson's Correlation Analysis. Data are expressed as mean ± SEM.

## Results

### Decline in brain glucose uptake and transporter expression at early stage of female aging

#### NonTg brain

**Figure 1:**
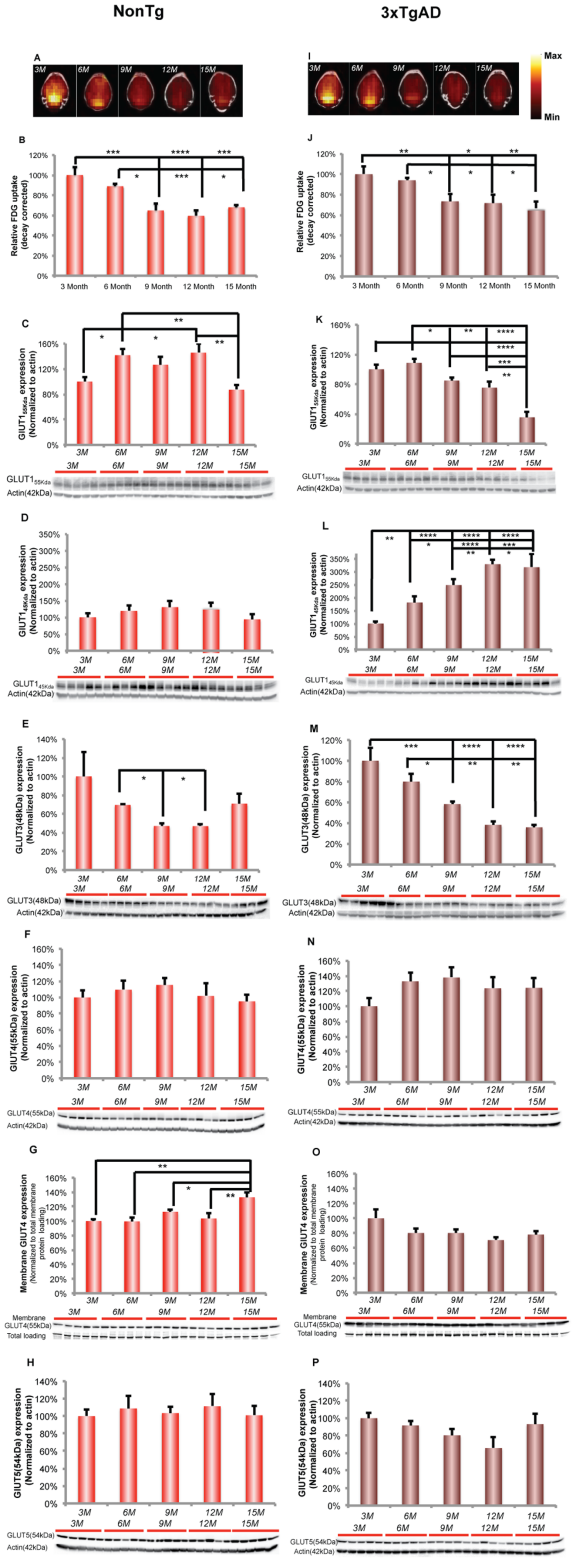
Decreased brain glucose uptake and glucose transporter expressions at early stage of female aging. A. Representative FDG-microPET images showed an age-related decline in brain glucose uptake in nonTg brain, which was maximal between 6 and 9 months of age. (Yellow indicates higher values and red indicates lower value). B. Quantitative analysis demonstrated an age-related decrease in brain glucose uptake in nonTg brain, which was significant between 6 and 9 months of age. C. GLUT1_55 Kda_ expression showed a significant increase after 3 months of age. This rise sustained across 6, 9 and 12 months of age and then decreased significantly at 15 months of age. D. In nonTg hippocampus, there was a trend towards increased expression of GLUT1_45 Kda_ from 3 to 12 months of age. However, it did not reach significance. E. GLUT3 expression in nonTg hippocampus demonstrated an age-related decline, which reached statistical significance between 6 and 9 months of age. F. There was no change in GLUT4 expression in nonTg hippocampus. G. Membrane GLUT4 expression increased significantly at 15 months of age. H. GLUT5 expression did not change in nonTg hippocampus. I. Representative FDG-microPET images showed an age-related decline in brain glucose uptake in 3xTgAD brain, which was maximal between 6 and 9 months of age. (Yellow indicates higher values and red indicates lower value). J. Quantitative analysis demonstrated an age-related decrease in brain glucose uptake in 3xTgAD mice, which was significant between 6 and 9 months of age. K. 3xTgAD hippocampus demonstrated an age-related decrease in the expression of GLUT1_55 Kda_, which was significant after 6 months of age. L. In 3xTgAD hippocampus, GLUT1_45 Kda_ increased significantly during aging. M. The age-related decline in GLUT3 expression reached significance after 6 months of age. N. GLUT4 expression did not change in 3xTgAD hippocampus. O. Membrane GLUT4 expression did not change in 3xTgAD hippocampus. P. There was no change in GLUT5 expression in 3xTgAD hippocampus. * p<0.05, ** p<0.01, *** p<0.001, **** p<0.0001, bars represent mean value ± SEM.

To investigate the impact of female aging on brain glucose metabolism, microFDG-PET/CT imaging was conducted to determine brain glucose uptake in female nonTg and 3xTgAD mice at 3, 6, 9, 12, 15 months of age. In nonTg mice, there was an age-related decline in brain glucose uptake (Fig. 1A), as indicated by semi-quantitative standard uptake value (SUV), which reached statistical significance between 6 and 9 months of age (Fig. 1B; F (4, 19) = 13.16, p<0.0001, n = 4–5/group). No evidence for recovery of glucose uptake was evident as the deficit in glucose uptake was sustained at 12 and 15 months of age.

The age-related decline in brain glucose uptake could be attributed to multiple levels of metabolic dysregulation, including reduced glucose transport, compromised glycolysis and deficient mitochondrial capacity [1], [3], [21], [22]. Based on the principle underlying FDG-PET imaging, 2-[^18^F]fluoro-2-deoxy-D-glucose (FDG) can be phosphorylated by hexokinase to FDG-6-phosphate. FDG-6-phosphate does not undergo further metabolism and is subsequently trapped in cells. Thus, the most direct limiting factors of microPET signal are the system of blood glucose/FDG supply (concentration), brain glucose/FDG transport, and hexokinase activity. In our study, because we injected the same sufficient amount of FDG, the blood FDG supply would not be the limiting factor.

To assess the impact of female aging on the expression of brain glucose transporters (GLUTs) in nonTg brain, we determined hippocampal protein expression of blood brain barrier (BBB) GLUT1_55 Kda_ (GLUT1_55 Kda_), glial GLUT1_45 Kda_ (GLUT1_45 Kda_), neuronal GLUT 3 (GLUT 3), neuronal GLUT4 (GLUT4, total and membrane fraction) and microglial GLUT5 (GLUT5) [23], [24].

In nonTg hippocampus, expression of the BBB GLUT1_55 Kda_ showed a significant increase after 3 months of age. The rise in GLUT1_55 Kda_ expression was sustained across 6, 9 and 12 months of age, followed by a significant decline at 15 months of age (Fig. 1C; F (4, 20) = 5.956, p<0.005, n = 5/group). The glial GLUT1_45 Kda_ showed a non-significant trend towards increasing from 3 to 12 months of age (Fig. 1D). GLUT3 expression demonstrated an age-related decline, which reached statistical significance between 6 and 9 months of age (Fig. 1E; F (4, 19) = 2.994, p<0.05, n = 4 – 5/group). In addition, expression of GLUT3 was significantly positively correlated with the decline in brain glucose uptake (Fig. 1E; Pearson's r = 0.89, p<0.05, n = 4–5/group). In addition to constitutively expressed glucose transporters, we investigated expression of GLUT4 which is induced by insulin signaling [25]. GLUT4 total protein expression did not change with age (Fig. 1F) whereas expression of GLUT4 in the membrane fraction increased significantly at 15 months of age (Fig. 1G; F (4, 20) = 6.531, p<0.005, n = 5/group). Microglial GLUT5 expression did not change significantly across different age groups (Fig. 1H).

#### 3xTgAD brain

In 3xTgAD mice, there was an age-related decline in brain glucose uptake (Fig. 1I), as determined by microFDG-PET and indicated by semi-quantitative standard uptake value (SUV), which reached statistical significance between 6 and 9 months of age (Fig. 1J; F (4, 18) = 7.084, p<0.005, n = 4–5/group). As with the nonTg brain, the deficit in brain glucose uptake was sustained at 12 and 15 months indicating no recovery at these ages.

To assess the impact of female aging on the expression of brain glucose transporters (GLUTs) in 3xTgAD brain, we determined the hippocampal protein expression of GLUT1_55 Kda_ (blood brain barrier (BBB) GLUT1_55 Kda_), GLUT1_45 Kda_ (glial GLUT1_45 Kda_) and GLUT 3 (neuronal GLUT 3), neuronal GLUT4 (GLUT4, total and membrane fraction) and microglial GLUT5 (GLUT5) [23], [24].

In 3xTgAD hippocampus, there was an age-related decline in GLUT1_55 Kda_, which reached significance at 9 months of age (Fig. 1K; F (4, 19) = 19.08, p<0.0001, n = 4–5/group). The decline in GLUT1_55 Kda_ at 9 months was maintained until 12 months followed by a precipitous decline evident at 15 months of age. The decline in GLUT1_55 KDa_ was positively correlated with the brain glucose uptake (Fig. 1K; Pearson's r = 0.83, p = 0.08, n = 4–5/group). Hippocampal glial GLUT_45 Kda_ expression underwent a significant rise in expression with age in 3xTgAD mice that was asymptotic at 12 months of age and maintained thereafter (Fig. 1L; F (4, 19) = 29.14, p<0.0001, n = 4–5/group). The rise in GLUT_45 Kda_ was negatively correlated with the brain glucose uptake (Fig. 1L; Pearson's r = −0.95, p<0.05, n = 4–5/group). In parallel, neuronal GLUT3 expression underwent an age-related decline, which was significant at 9 months of age (Fig. 1M; F (4, 19) = 16.08, p<0.0001, n = 4–5/group). Further, expression of GLUT3 was significantly positively correlated with brain glucose uptake (Fig. 1M; Pearson's r = 0.96, p<0.001, n = 4–5/group). Neither Expressions of GLUT4 (both total and membrane) nor GLUT5 changed during female aging (Fig. 1N, Fig. 1O and Fig. 1P).

### Decline in glycolytic capacity at early stage of female aging

Hexokinase irreversibly phosphorylates glucose to glucose-6-phosphate, which is the first and rate-limiting step in glycolysis. The FDG uptake signal in FDG-PET imaging positively correlates with hexokinase activity, as cellular FDG accumulation is mediated by hexokinase that phosphorylates FDG to FDG-6-phosphate, which does not undergo further metabolism [26]. To investigate whether the decline in brain glucose uptake is associated with compromised glucose phosphorylation, we analyzed the activity of hexokinase and expression of hexokinase type II, the specific isozyme sensitive to hormonal regulation [27]. The key enzyme linking aerobic glycolysis to oxidative phosphorylation is pyruvate dehydrogenase (PDH). Phosphorylation of the E1 subunit of PDH inactivates E1 that results in activation of the entire PDH complex. PDHα has 3 phosphorylation sites, phosphorylation of site 1 (Ser293) reduces overall PDC activity by>97% [28]. To investigate the impact of female aging on PDH inactivation, we analyzed the phosphorylation level of Ser293 on PDH protein.

#### NonTg brain

**Figure 2:**
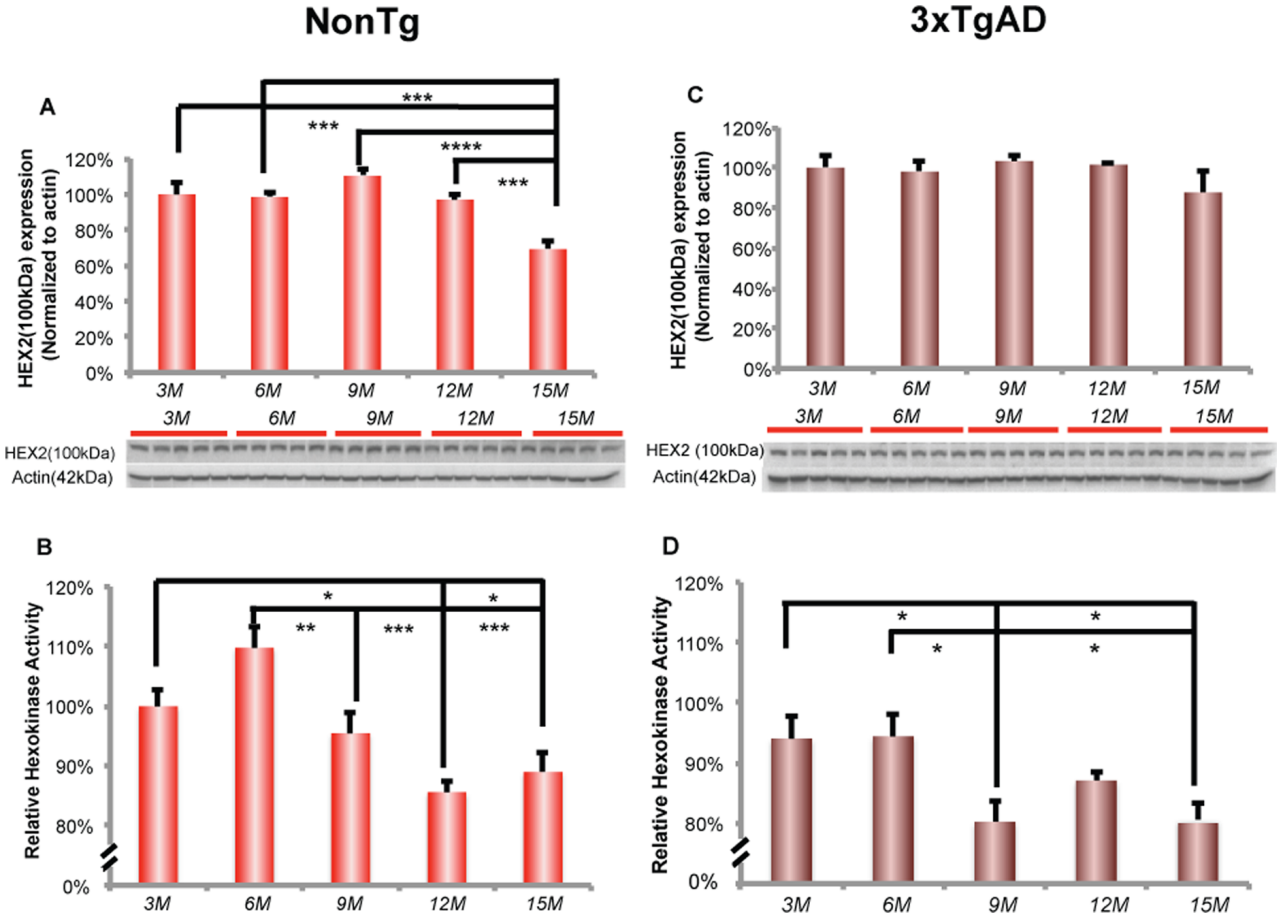
Decreased hexokinase activity at early stage in female aging. A. In nonTg hippocampus, expression of hexokinase 2 decreased significantly at 15 months of age. B. Hexokinase activity in nonTg hippocampus decreased significantly after 6 months of age. C. There was no change in the expression of hexokinase 2 in 3xTgAD hippocampus. D. Hexokinase activity in 3xTgAD hippocampus decreased significantly after 6 months of age. * p<0.05, ** p<0.01, *** p<0.001, **** p<0.0001, bars represent mean value ± SEM.

In nonTg mice, hippocampal hexokinase II protein expression decreased significantly at 15 months of age (Fig. 2A; F (4, 20) = 12.79, p<0.0001, n = 5/group), whereas hippocampal hexokinase activity was significantly decreased earlier at 9 months of age (Fig. 2B; F (4, 20) = 9.775, p<0.0005, n = 5/group). Further, the decline in hexokinase activity was sustained at the decreased level across 9, 12 and 15 months of age. The decline in hexokinase activity was positively correlated with brain glucose uptake (Fig. 2B; Pearson's r = 0.77, p = 0.13, n = 5/group).

**Figure 3:**
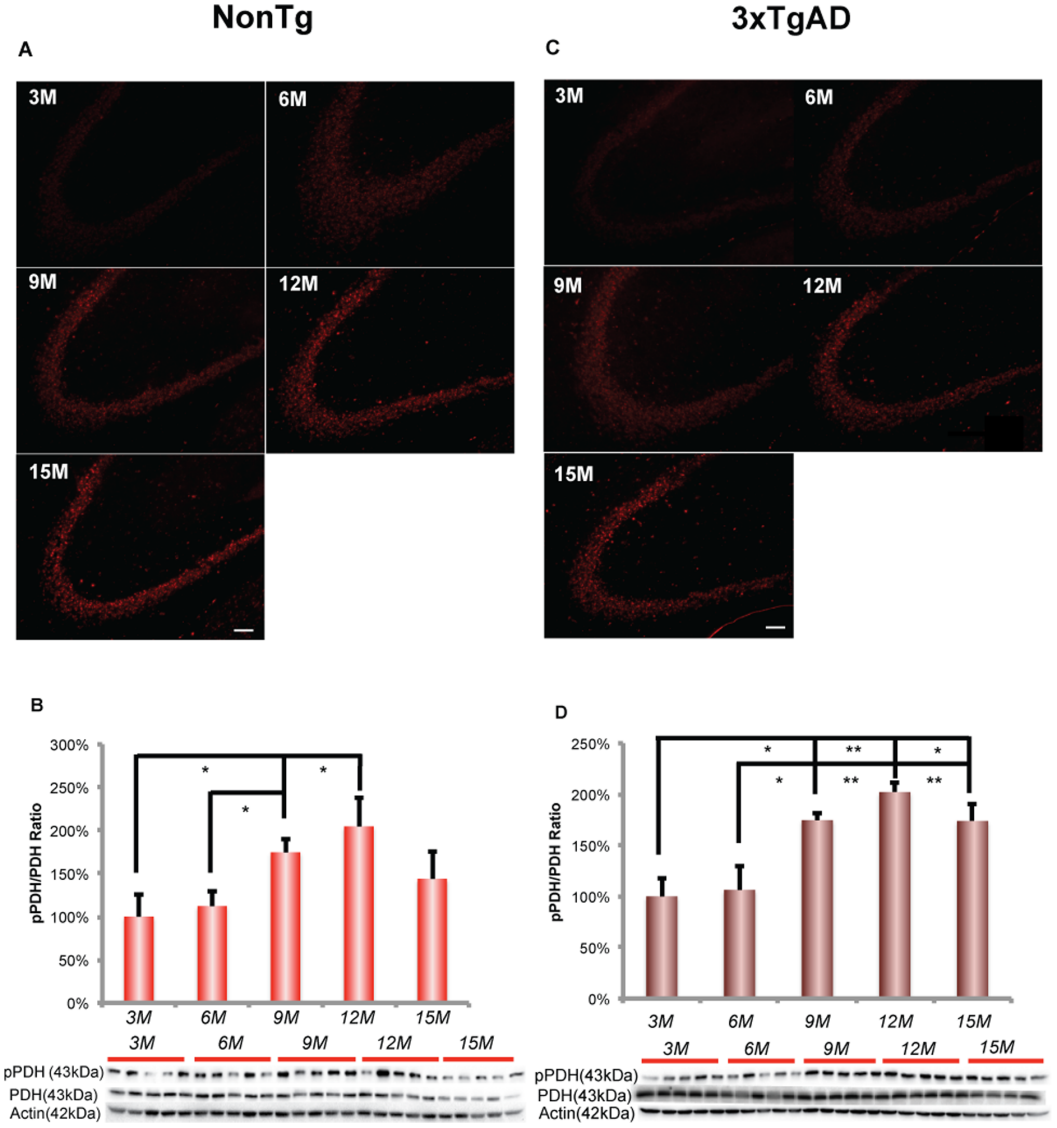
Increase in the phosphorylation of PDH at early stage of female aging. A. Immunofluorescent labeling of phosphoPDH (Ser293) in the nonTg hippocampal CA3. B. NonTg hippocampus demonstrated an age-related increase in phosphoPDH/PDH ratio, which was significant after 6 months of age. C. Immunofluorescent labeling of phosphoPDH (Ser293) in the 3xTgAD hippocampal CA3. D. 3xTgAD hippocampus demonstrated an age-related increase in phosphoPDH/PDH ratio, which was significant after 6 months of age. * p<0.05, ** p<0.01, bars represent mean value ± SEM. Scale: 100 µm.

In parallel to the decline in hexokinase activity, immunofluorescent phosphoPDH (Ser293) increased dramatically in the hippocampal CA3 region (Fig. 3A). Increased phosphoPDH was detectable at 6 and 9 months of age and increased at 12 and 15 months of age. Expression of phosphoPDH (Ser293) protein relative to total PDH expression exhibited an age-related increase, which reached significance at 9 and 12 months of age (Fig. 3B; F (4, 18) = 4.002, p<0. 05, n = 4–5/group). Further, the phosphorylated state of PDH declined at 15 months. PDH phosphorylation was significantly negatively correlated with brain glucose uptake in nonTg mice (Fig. 3A; Pearson's r = −0.93, p<0.05, n = 4–5/group).

#### 3xTgAD brain

In 3xTgAD hippocampus, hexokinase II protein expression did not change with age (Fig. 2C). However, hexokinase activity significantly declined with age, which was significant at 9 months of age (Fig. 2D; F (4, 19) = 4.561, p<0.001, n = 4–5/group) and was sustained at decreased level at later ages of 12 and 15 months. Moreover, the decline in hexokinase activity was significantly positively correlated with brain glucose uptake (Fig. 2D; Pearson's r = 0.91, p<0.05, n = 4–5/group).

In 3xTgAD hippocampus, there was an increased level of immunofluorescent phosphoPDH (Ser293) signal in hippocampal CA3 region (Fig. 3C). PhosphoPDH/PDH protein expression ratio exhibited an age-related increase, which was significant at 9 months of age (Fig. 3D; F (4, 20) = 7.956, p<0. 001, n = 5/group) and sustained at increased level at later ages of 12 and 15 months. Moreover, the increased phosphoPDH/PDH protein expression increase was significantly negatively correlated with brain glucose uptake (Pearson's r = −0.94, p<0.05, n = 4–5/group).

### Decline in lactate generation and utilization at early stage of female aging

A decline in brain glucose utilization and associated metabolic pathways should induce mechanisms to compensate for the decline in glucose. As we and others have shown, neurons can utilize lactate or ketone bodies as alternative fuels [2], [29]–[31]. In response to immediate energetic demand, brain can utilize lactate to sustain synaptic transmission, whereas under prolonged glucose deprivation, the brain will utilize ketone bodies generated from the liver to support the energetic demand [32]. Thus, lactate utilization would be the first adaptive response to support brain energy demand [2], [29], [33]. To investigate whether the decline in brain glucose uptake is associated with a shift in utilization of lactate, we first determined the expression of lactate dehydrogenase (LDH) protein in nonTg and 3xTgAD female mice of 3, 6, 9, 12 and 15 months of age. Glial and neuronal cells have different LDH isoforms. LDH5 is the major isoform expressed in glial cells and converts pyruvate to lactate to generate lactate whereas LDH1 is the major isoform in neurons and functions to convert lactate to pyruvate thereby providing an indicator of lactate utilization. The ratio of LDH5/LDH1 ratio provides an indicator of whether the two systems are functioning in a coordinated manner or whether there is a dysregulation between the system of generation and utilization of lactate predictive of lactate accumulation.

#### NonTg brain

**Figure 4:**
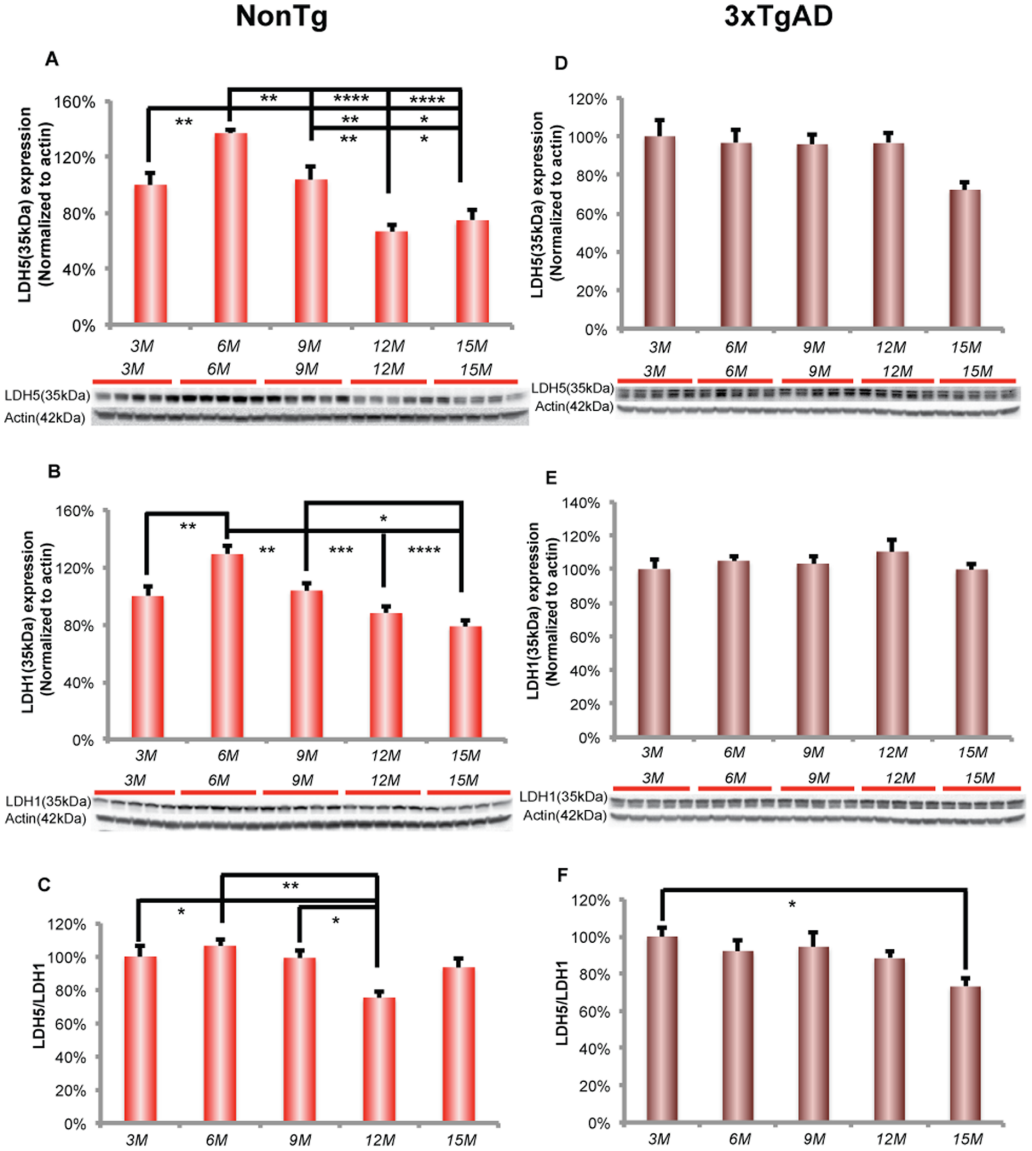
Reproductive transition paralleled a significant decrease in LDH5 and LDH1 expressions in nonTg hippocampus. A. NonTg hippocampus demonstrated a an age-related decrease in LDH5 expression from 6 to 15 months of age, which was significant between 6 and 9, 9 and 12 months of age. B. LDH1 expression decreased with age in nonTg hippocampus from 6 to 15 months of age, which was significant between 6 and 9, 9 and 15 months of age. C. LDH5/LDH1 ratio decreased significantly at 12 months of age. D. There was no change in 3xTgAD LDH5 expression. E. There was no change in 3xTgAD LDH1 expression. F. LDH5/LDH1 ratio decreased significantly at 15 months of age. * p<0.05, ** p<0.01, *** p<0.001, **** p<0.0001, bars represent mean value ± SEM.

In nonTg hippocampus, an age-related decrease in glial LDH 5 protein expression was apparent at 9 months of age and was sustained across 12 and 15 months of age (Fig. 4A; F (4, 20) = 15.89, p<0.0001, n = 5). In parallel, neuronal LDH1 exhibited a comparable pattern of age-related decline beginning at 9 months, which was sustained at the decreased level at later ages of 12 and 15 months (Fig. 4B; F (4, 20) = 12.72, p<0.0001, n = 5). The decline in both LDH5 and LDH1 suggest that lactate production and utilization decreased with aging. Moreover, LDH5/LDH1 ratio decreased significantly at 12 months of age (Fig. 4C; F (4, 20) = 6.006, p<0.01, n = 5). LDH5 and LDH1 expression was significantly positively correlated with glycolytic enzymes, especially with hexokinase activity (Fig. 4A (LDH5); Pearson's r = 0.97, p<0.005, n = 5/group; Fig. 4A (LDH1); Pearson's r = 0.92, p<0.05, n = 5/group). The parallel decline in both glucose and lactate pathways suggested a coordinated regulation of glycolytic capacity and lactate generation/utilization. The decline of LDH5 and LDH1 expression in nonTg mice indicates that lactate is unlikely to be the alternative fuel to compensate brain glucose hypometabolism during female aging process in nonTg brain.

#### 3xTgAD brain

In 3xTgAD hippocampus, there was no change at any age in LDH5 and LDH1 expression (Fig. 4D and Fig. 4E). However, the ratio of LDH5/LDH1 decreased significantly at 15 months of age (Fig. 4F; F (4, 20) = 3.365, p<0.05, n = 5).

### Activation of ketogenic pathway at early stage of female aging

In the nonTg brain, lactate is unlikely to be an alternative fuel to compensate for the decline in glucose metabolism, we therefore investigated whether ketone bodies were generated and utilized during female brain aging. Our previous analyses demonstrated that the key enzyme in ketone body metabolism, 3-oxoacid-CoA transferase (SCOT), increased with reproductive senescence [2], suggesting that ketone bodies are a potential alternative fuel. To determine whether a shift in utilization of ketone bodies paralleled the decline in brain glucose uptake, we first determined ketone body level in plasma followed by analysis of monocarboxylate transporters (MCTs) for transporting lactate/ketone bodies.

#### NonTg brain

**Figure 5:**
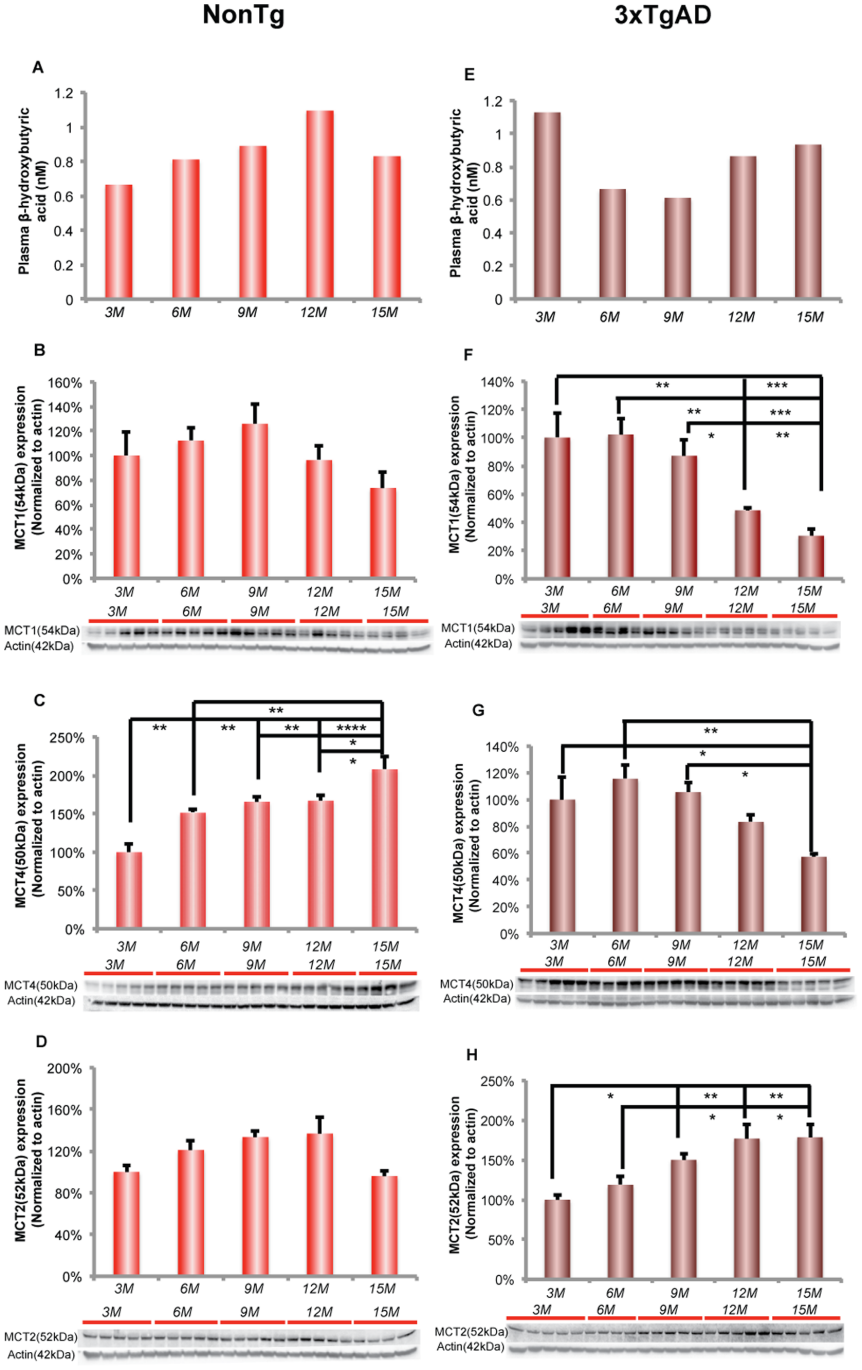
Plasma β-hydroxybutyrate level and its neuronal transporter increase with reproductive transition. A. Plasma β-hydroxybutyrate level increase with age in nonTg mice (plasma samples were pooled from 5 different animals, n = 1–2). B. MCT1 expression showed a trend towards increasing from 3 to 9 months of age and decreased afterwards in nonTg hippocampus. C. The age-related decrease in MCT4 expression was significant between 3 and 6, 6 and 15 months of age. D. MCT2 increased significant from 3 to 12 months with age (linear regression: slope = 0.07, R^2^ = 0.3273, p<0.05). E. In 3xTgAD mice, plasma β-hydroxybutyrate level was highest at 3 months of age. The β-hydroxybutyrate level decreased after 3 months and increased again after 9 months of age (plasma samples were pooled from 5 different animals, n = 1–2). F. In 3xTgAD hippocampus, MCT1 expression showed an age-related decline, which was significant at 12 and 15 months of age. G. The age-related decrease in MCT4 expression was significant at 15 months of age. H. The age-related increase in MCT2 expression was significant between 6 and 9 months of age. * p < 0.05, ** p < 0.01, *** p < 0.001, **** p < 0.0001, bars represent mean value ± SEM.

In pooled nonTg plasma samples (n = 5), β-hydroxybutyrate level increased with age and reached maximum at 12 months of age. Plasma β-hydroxybutyrate level was negative ely correlated with brain glucose uptake level (Fig. 5A; Pearson's r = −0.85, p = 0.06).

MCTs transport monocarboxylates such as lactate, pyruvate and ketone bodies across the cell membrane. MCT1 is specifically expressed in glial cells and at the blood brain barrier (BBB) whereas MCT4 is expressed in astrocytes. MCT2 is mainly expressed in neurons and has been found in cell bodies and in postsynaptic densities [33], [34]. To determine the ability of the brain to transport alternative substrates, we assessed protein expression level for BBB/glial MCT1, glial MCT4 and neuronal MCT2.

In nonTg hippocampus, BBB/glial MCT1 expression exhibited no significant change with age (Fig. 5B). In contrast, astocytic MCT4 expression significantly increased with age and was maximal at 15 months of age (Fig. 5C; F (4, 18) = 12.51, p < 0.0001, n = 4–5/group). There was a significant trend towards increase in neuronal MCT2 expression with age (Fig. 5D; F (4, 19) = 3.555, p<0.05, n = 4–5/group).

#### 3xTgAD brain

In pooled 3xTgAD plasma samples (n = 5), β-hydroxybutyrate concentration at 3 months of age was the highest among all 3xTgAD age groups, indicating an early ketogenic phenotype in 3xTgAD female mouse, which is consistent with our earlier findings [2]. The β-hydroxybutyrate concentration declined after 3 months of age and increased again 12 and 15 months of age (Fig. 5E).

Expression of BBB/glial MCT1 and astrocytic MCT4 in 3xTgAD hippocampus showed an opposite profile to that of neuronal MCT2. BBB/glial MCT1 expression decreased significantly at 12 and 15 months of age whereas significant decline in astrocytic MCT4 occurred later at 15 months of age (Fig. 5F (MCT1); F (4, 18) = 10.45, p<0.0005, n = 4–5/group; Fig. 5G (MCT4); F (4, 19) = 5.079, p<0.01, n = 4 – 5/group). In contrast, neuronal MCT2 increased significantly after 9 months of age and remained significantly elevated at 12 and 15 months of age (Fig. 5H; F (4, 19) = 7.29, p<0.005, n = 4−5/group). The opposing pattern of MCT1/MCT4 and MCT2 expression suggest a disconnection between BBB/glial transport of alternative fuel supply and the alternative fuel requirements from neurons.

## Discussion

In this study, we sought to determine whether 1) the system of brain fuel supply and transport changed with aging in the female brain and 2) whether the changes in this vital system were antecedent to or subsequent to the decline in mitochondrial function that we had previously observed [2], [4]. Results of analyses reported herein indicated that 1) decline in brain glucose uptake occurred early in the process of female brain aging; 2) decline in brain glucose uptake is paralleled by decline in glucose transporter expression in neurons and rise in glial associated glucose transporters; 3) decline in brain glucose uptake and neuronal glucose transporter expression are paralleled by decline in key metabolic enzymes required for glucose metabolism; 4) decline in glucose metabolism is paralleled by increases in alternative substrate supply (ketone bodies) and associated transporters (MCT2 and MCT4); and lastly 5) these changes in brain glucose supply and the system required for glucose transport and metabolism precede development of mitochondrial dysfunction. These changes in the systems required for substrate supply for production of ATP to support energetic demand temporally mapped onto the earliest phase of reproductive transition (6–9 months). The decline in brain glucose uptake and neuronal glucose transport preceded the shift to alternative, less efficient fuels, which coincided with the age of reproductive senescence (9–12 months).

Results of analyses of the aging female nonTg brain demonstrated a significant decline in brain glucose uptake at an early stage in female brain aging. The decline in brain glucose uptake, indicated by microFDG-PET imaging, could be attributed to multiple levels of metabolic dysregulation, including reduced glucose transport, compromised glycolysis, deficient mitochondrial capacity and/or impaired ATP generation. In the nonTg brain, blood-brain-barrier (BBB) GLUT1_55 Kda_ increased significantly relative to 3-month-old female hippocampus then declined at 15 months. In parallel to the significant rise in BBB GLUT1_55 Kda_, there was a trend toward increased glial GLUT1_45 Kda_ expression. Coincident with the rise in BBB GLUT1_55 Kda_ and glial GLUT_45 Kda_ transporters, neuronal GLUT3 decreased over the same time frame and then rebounded at 15 months. GLUT3 expression coupled to metabolic demand or cerebral glucose utilization [35]–[37]. In the aging female brain, the pattern of glucose uptake and glucose transporter expression is consistent with the decrease in microFDG-PET signal and thus largely driven by the decline in neuronal glucose uptake via GLUT3. No significant change occurred in GLUT4 and GLUT5 expression preceding or during reproductive transition, suggesting that insulin-dependent neuronal uptake or microglia uptake of glucose did not contribute to the decline in microPET signal. Although in nonTg brain, a significant increase in GLUT4 occurred at 15 months but did not reverse the significant decrease in whole brain FDG-PET. Collectively, the data indicate that decline in microFDG-PET signal and brain metabolic activity is largely determined by neuronal glucose transport and metabolism.

In contrast to the decline in neuronal glucose transporter, an adaptive rise in BBB GLUT1_55 KDa_ and glial GLUT1_45 Kda_ expression occurred which likely represents a compensatory strategy to provide glucose to the brain. However, the rise in these non-neuronal glucose transporters did not alter the sustained deficit in microFDG-PET signal. GLUT1_45 Kda_ is mainly expressed in oligodendrocytes and astrocytes with localization that includes the astrocytic endfeet adjacent to the BBB endothelial cell [24], [38]. GLUT1_45 Kda_ could increase glucose uptake into astrocytes, where glucose could be converted to lactate or into glycogen to serve as a compensatory fuel for neurons. However, the decline in both LDH5 to generate lactate in astrocytes and LDH1 in neurons to utilize lactate makes this compensatory option unlikely. Further, the rise in phosphorylated/inactivated PDH compromises the utilization of lactate by neurons as lactate is converted to pyruvate, which requires active PDH for conversion to acetyl-CoA for entry into the TCA cycle. Alternatively, GLUT1_45 Kda_ could increase glucose transport into oligodendrocytes to address energetic demands of lipid/myelin synthesis [39], [40]. Together, the compensatory rise in GLUT1_45 Kda_ is more likely associated with transport of glucose for lipid/myelin synthesis [41].

Cerebral glucose utilization is also controlled by hexokinase, which is the first rate-limiting step in glycolysis and is critical in mediating the FDG-PET signal [26]. We found that a decline in hippocampal hexokinase activity paralleled the decline in brain glucose uptake and GLUT3 expression. Compromised glucose transport and hexokinase activity could be further exacerbated by decreased activity of PDH, which converts pyruvate to acetyl-CoA and is the key enzyme linking glycolysis and TCA cycle. Coincident with the decline in glucose uptake, GLUT3 expression and hexokinase activity, we observed an age dependent increase in phosphorylated PDH, a mechanism of PDH inactivation. Taken together, these data are indicative of a system-wide deficit, from transport to cerebral glucose metabolism in the hippocampus at an early stage of female brain aging that precedes both reproductive senescence and the associated decline in mitochondrial function.

The 3xTgAD brain also exhibited an age-related decline in microFDG-PET that occurred between 6 and 9 months of age. In parallel to the decline in brain glucose uptake, expression of the blood-brain-barrier (BBB) GLUT1_55 Kda_ and neuronal GLUT3 also decreased. The decline in BBB GLUT1_55 Kda_ is consistent with earlier reports of reduced BBB glucose transporter in postmortem AD cerebral cortex [42]. The inability of the 3xTgAD brain to mount a compensatory rise in GLUT1_55 Kda_ expression may be related to accumulation of vascular β-amyloid, which has been reported to impair BBB GLUT1_55 Kda_ expression [43]. Further, the decline in both glucose uptake and hippocampal GLUT1_55 Kda_ expression in the female 3xTgAD brain is consistent with our previous findings that ovariectomy induced a significant decline in both these indicators of brain glucose metabolism [44]. Further, the β-amyloid mechanism of reduced GLUT1_55 Kda_ expression is consistent with the age-related and ovariectomy-associated increase in β-amyloid deposition in the 3xTgAD brain [3], [4]. In stark contrast to the decline in GLUT1_55 Kda_, there was a dramatic rise in glial GLUT1_45 Kda_ expression. The significant rise in GLUT1_45 Kda_ was unique to the 3xTgAD hippocampus although a similar trend was observed in the nonTg hippocampus.

Under normal conditions, glucose transport across the BBB is not considered to be rate limiting [45], [46]. However, under pathological conditions BBB glucose transport can be a rate-limiting step in brain metabolism, leaving neurons and glial cells vulnerable to glucose deprivation [47]. This appears to be the case in the 3xTgAD female brain. The pattern of a concomitant decline in BBB and neuronal glucose transporters in parallel to a dramatic rise in the glial GLUT1_45 Kda_ glucose transporter, present in oligodendrocytes and the endfeet of astrocytes, suggests either: 1) a compensatory upregulation of astrocytic endfeet glucose transporters to compensate for the decline in glucose transport through the BBB or 2) preferential transport of glucose into oligodendrocytes to meet the energetic demand of white matter generation and maintenance [39], [48].

In addition to significant changes in glucose transporter expression, the 3xTgAD brain also exhibited a significant decrease in hippocampal hexokinase activity at an early stage in female aging. This age-associated decline is consistent with our previous finding that ovarian hormone loss in 3xTgAD mice induced a significant decline in hexokinase activity, which was partially prevented by 17β-estradiol treatment [44]. Compared to nonTg mice, 3xTgAD mice exhibited a lower hippocampal hexokinase activity at each age. Of note, β-amyloid in the 3xTgAD brain is detectable in the mitochondria at 9 months of age and thereafter [4], and is temporally coincident with a significant decline in hexokinase activity 3xTgAD hippocampus [44]. The decline in hexokinase activity and the appearance of β-amyloid in mitochondria are consistent with previous *in vitro* assays in cultured neurons demonstrating that β-amyloid triggers the release of neuronal hexokinase 1 from mitochondria and inactivation of hexokinase 1 [49]. The decline in BBB and neuronal glucose transporters coupled with decreased hexokinase activity was accompanied by increased phosphorylation of PDH, consistent with decreased PDH activity. We found that in 3xTgAD hippocampus, there was an increase in the phosphorylation of the PDH complex at the early stage of female aging, which is consistent with previous findings of reproductive senescence-associated and ovariectomy-induced decline in PDH activity [2], [3].

Collectively, these data indicate a system-wide decline in glucose metabolism evidenced in glucose uptake into the brain, glucose transporters and glucose metabolism. Further, the data show that the glucose transporter system in hippocampus is dynamic in its compensatory response potential with the nonTg and 3xTgAD brains generating different adaptive responses. The nonTg brain favors a strategy to increase glucose transport across the blood brain barrier while lowering glucose availability to energetically demanding neurons in favor of a modest increase of glucose transport into glial cells, either oligodendrocyte or astrocytic endfeet. In contrast, the 3xTgAD brain favors a glial compensatory response to increase glucose transport either into oligodendrocyte or astrocytic endfeet.

In response to decreased glucose uptake, the brain can utilize lactate or ketone bodies as alternative fuels. Lactate is known to be a metabolic substrate for brain and is particularly important under conditions of high synaptic activity, which occurs during learning and memory [50]. Further, during glucose insufficiency, lactate can serve as a secondary fuel through glycogen metabolism [34]. In nonTg mice, expression of LDH5 and LDH1 decreased during aging, suggesting decreased generation and utilization of lactate in nonTg hippocampus during female aging. The decrease in LDH5/LDH1 ratio also suggested that there was less lactate accumulation in nonTg hippocampus during female aging. In nonTg brain, the pattern of decreasing LDH5 and LDH1 expression is similar to the decreasing pattern of the system required for glucose transport and metabolism, indicative of coordinated regulation between the glucose and lactate systems which is consistent with glucose as the metabolic precursor to lactate in aerobic glycolysis for conversion of pyruvate to lactate via LDH5. The parallel decline in glucose uptake and LDH isoforms 5 and 1, predict that astrocyte-derived lactate would not serve as an alternative fuel source to compensate for the decline in glucose availability. However, transport of lactate from the periphery remains a possibility, as BBB/glial MCT1 did not decline until 15 months of age and the astrocytic MCT4 significantly increased with age. However, the rise in phosphorylated/inactivated PDH compromises the utilization of lactate by neurons as lactate is converted to pyruvate, which requires active PDH for conversion to acetyl-CoA for entry into the TCA cycle.

In 3xTgAD mice, expression of LDH5 and LDH1 did not change during the aging process, suggesting a lack of response in lactate compensatory system and loss of dynamic adaptation during 3xTgAD female brain aging. The findings in lactate generation/utilization system in 3xTgAD hippocampus were in contrast to our earlier findings that in the ovariectomized female 3xTgAD mouse hippocampus, expression of LDH isoforms 5 and 1 along with the LDH5/LDH1 ratio were increased [44]. These, data indicate an essential difference between the gradual loss of ovarian hormone homeostasis during 3xTgAD female brain aging and an acute loss of ovarian hormones due to surgical ovariectomy. The sustained expression of LDH5/LDH1 throughout the aging process suggests that in the 3xTgAD hippocampus, the tight coupling between glucose and lactate metabolic systems that characterizes the nonTg hippocampus is not expressed in the 3xTgAD hippocampus. Further, the data suggest that transport of lactate from the periphery into the astrocytes and oligodendrocytes would be compromised by the dramatic decline in MCT1 expression at 12 and 15 months and the concomitant decline in MCT4 during the same period. The dramatic rise in neuronal MCT2, when BBB and glial MCTs are decreasing, suggest a disconnection between BBB/glial transport of alternative fuel supply and the alternative fuel requirements from neurons. Although lactate could be provided to the brain from the periphery, the rise in phosphorylated/inactivated PDH comprises the utilization of lactate in neurons as lactate requires conversion to pyruvate which in turn requires active PDH for conversion to acetyl-Co-A for entry into the TCA cycle. Together, the data suggest that lactate could serve as an alternative fuel in some cells but that in hippocampus utilization of lactate will be limited in magnitude.

In addition to lactate, ketone bodies can also serve as an alternative fuel for brain during early development and starvation [51]. In the current study, we found that in the nonTg mouse, plasma concentration of the ketone body, β-hydroxybutyrate, increased in parallel with the decline in glucose uptake. There was no change in the BBB MCT1 and only a modest rise in neuronal MCT2 expression until 12 months of age. In contrast, astrocytic MCT4 expression increased significantly at 15 months. Although neuronal MCT2 and astrocytic MCT4 are actively involved in lactate transport [33], [52], increased MCT2/MCT4 expression in nonTg aging hippocampus also has the potential to increase ketone body transport. The rise in astrocytic MCT4 is consistent with our previous findings that reproductive senescence is associated with activation of the ketogenic pathway indicated by an increase in the rate limiting enzyme for ketone body utilization, 3-oxoacid-CoA transferase (SCOT) [2]. The rise in astrocytic MCT4, together with the earlier findings, are indicative of a compensatory response in the aging female brain to increase systems required for generation, metabolism and transport ketone bodies in response to the decline in glucose uptake [2].

In 3xTgAD mice, plasma β-hydroxybutyrate concentration was maximal at an early age (3 months of age), decreased at 6 and 9 months of age, and rose again at 12 and 15 months of age. The elevation of peripheral ketone bodies at 3 months of age is consistent with our previous findings that hippocampal SCOT expression which is required for catabolism of ketone bodies to generate acetyl-CoA [2]. In 3xTgAD brain, the early activation of ketogenic pathway was also evident as an increase in gene expression for enzymes involved in ketone body metabolism at 6 months of age [53]. Consistent with the maximal level of plasma β-hydroxybutyrate, expression of MCT1 and MCT4 were highest at 3 months of age, sustained until 9 months of age and decreased dramatically thereafter. The decline in MCT1 and MCT4 at 12 and 15 months would limit the transport of ketone bodies into astrocytes and oligodendrocytes. However, the rise in neuronal MCT2 would preferentially transport either lactate and or ketone bodies into neurons. We and others have documented that neurons can utilize ketone bodies as an alternative fuel [2], [54].

**Figure 6:**
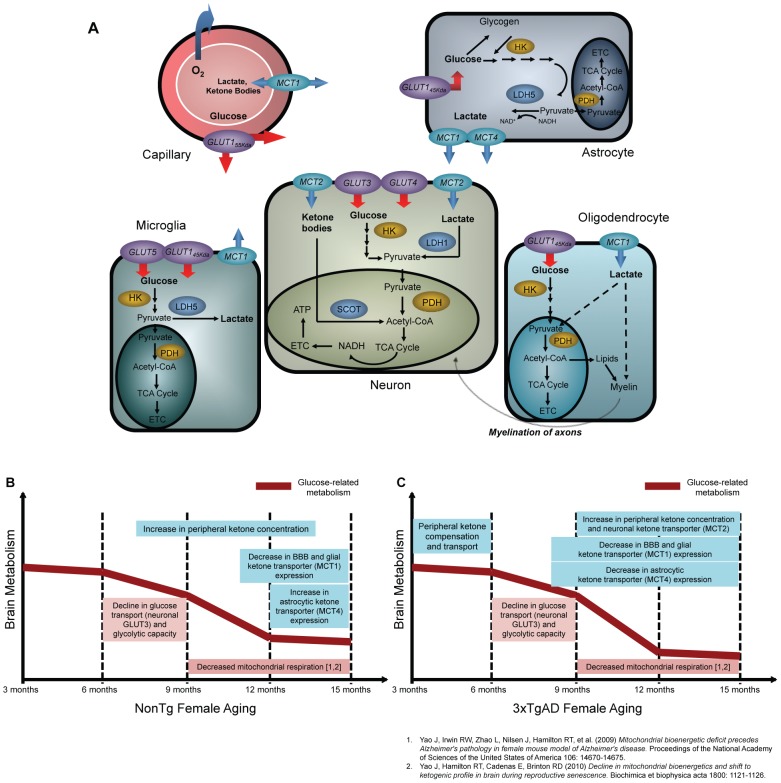
Schematic of brain bioenergetics and timeline of bioenergetic aging in female mammalian brain. A. In neurons, glucose is transported through the blood-brain-barrier glucose transporter 1 (GLUT1_55 Kda_), transported into neurons by glucose transporter 3 (GLUT3), phosphorylated by hexokinase and further metabolized into pyruvate. Pyruvate is converted to acetyl-CoA by pyruvate dehydrogenase to enter into TCA cycle to generate ATP. In astrocytes, glucose is transported by glucose transporter 1 (GLUT1_45 Kda_) and phosphorylated by hexokinase to generate pyruvate, which can be converted to lactate by lactate dehydrogenase 5 (LDH5). In oligodentrocytes, glucose is transported into oligodendrocyte to generate ATP or serve as carbon skeleton in lipid/myelin synthesis. In microglia, glucose is transported by GLUT5 (mainly) and GLUT1_45 Kda_ for downstream metabolic pathways. The alternative fuels, lactate or ketone bodies, are transported through blood-brain-barrier monocarboxylate transporter 1 (MCT1). Lactate generated by astrocyte is transported by MCT1 or MCT4. Lactate and ketone bodies are transported by MCT2 into neuron, where lactate is converted to pyruvate by lactate dehydrogenase 1 (LDH1) and ketone bodies is converted to acetyl-CoA by 3-oxoacid-CoA transferase (SCOT) to generate ATP. In oligodendrocyte, lactate can be transported by MCT1 and is suggested to serve important role in energy production and lipid/myelin synthesis. In microglia, lactate is generated by LDH5 and transported by MCT1. B. In normal nonTg brain, the decline in brain glucose transport and glycolytic capacity occurred between 6 and 9 months of age, which temporally preceded mitochondrial dysfunction. The decline in brain bioenergetic was paralleled with the increase in peripheral ketone body concentration. The expression of BBB and glial ketone body transporter decreased after 9 months of age whereas the astrocytic ketone body transporter increased at 15 months of age. C. In 3xTgAD brain, the decline in brain glucose transport and glycolytic capacity also occurred between 6 and 9 months of age, which temporally preceded mitochondrial dysfunction. The activation of the ketogenic pathway occurred at both early age and early stage of female aging. The expression of BBB/glial and astrocytic ketone transporters was maximal at early age (3 months) but decreased at early stage of female aging (9 to 15 months).

We previously demonstrated a significant age-related decline in aerobic glycolysis and mitochondrial respiration in nonTg female brain between 9 and 12 months of age [2], [4]. Findings from the current study demonstrate that the decline in brain glucose uptake, neuronal glucose transporter expression and glycolytic capacity in the aging female brain temporally preceded the decline in mitochondrial respiration [2], [4]. Further, the decline in brain glucose bioenergetics was paralleled by a shift toward ketone body utilization, a metabolic phenotype that precedes memory impairment, which is characteristic of early Alzheimer's disease (Fig. 6B) [2], [4], [55].

The 3xTgAD brain exhibited early activation of the ketogenic pathway and a lack of adaptive response during female aging, indicated by a decline in alternative fuel transport system (Fig. 6C). Compared to 3xTgAD brain, the nonTg brain has a more robust compensatory and dynamic adaptive system in terms of alternative fuel supply and transport. However, because mitochondria are metabolizing glucose, lactate and ketone bodies to produce ATP, both nonTg and 3xTgAD brain reach a stable state of diminished bioenergetic capacity after collapse of mitochondrial function.

Ovarian hormones are known to enhance or maintain brain glucose metabolism by increasing the expression of glucose transporters, and key rate limiting processes required for aerobic glycolysis and oxidative phosphorylation [21], [56]. In primate cerebral cortex 17β-estradiol increased the mRNA expression of GLUT1 and GLUT3 [57]. In addition to glucose transporters, estradiol and progesterone significantly increased hexokinase activity in aged female rat brain [58]. Further, ovarian hormone deprivation induced a significant decline in PDH activity, which was prevented by 17β-estradiol treatment [3].

Together, these findings provide a sequence of age-related events that are first evident as glucose hypometabolism and a shift to alternative fuels followed by mitochondrial dysfunction [2], [4]. Glucose hypometabolism appears to be the first and defining step in female brain aging, followed by mitochondrial dysfunction, leads to a bioenergetic phenotype consistent with the bioenergetic phenotype of persons at risk for AD [9], [13], [59], [60]. From a translational perspective, these findings suggest development of strategies that 1) prevent transition to bioenergetic deficits and 2) target fuel and enzyme systems of a brain that was transitioned to alternative fuels.
